# Supplementary material for: ProAgio, a Novel Integrin αvβ3 Targeted Cytotoxin, Suppresses Tumor Growth and Reprograms the PDAC Microenvironment
Source: bioRxiv. 2026 Jan 16:2026.01.15.699725. Preprint. [Version 1] doi: 10.64898/2026.01.15.699725 (PMC12871271; doi:10.64898/2026.01.15.699725)
Supplement: 1 [file NIHPP2026.01.15.699725V1-supplement-1.pdf]

bioRxiv preprint doi: https://doi.org/10.64898/2026.01.15.699725; this version posted January 16, 2026. The copyright holder for this preprint (which was not certified by peer review) is the author/funder, who has granted bioRxiv a license to display the preprint in perpetuity. It is made available under aCC-BY 4.0 International license.

Supplementary figure legends:

Supplementary Figure 1: Effect of GPH plus ProAgio on $CD8^+$ T cell subpopulations

Bar graphs represent the percentage changes in $CD8^+$ T cells **(A)**, $CD8^+$ CTLA-4^+ (B), $CD8^+$ PD-1^+ **(C)**, and $CD8^+$ CTLA-4^+ PD-1^+ T cells **(D)**. **(E)**. Expressions of $CD8^+$ subset populations, including naïve, pre-effector, effector, and central memory T cells, in vehicle, ProAgio, GPH, and GPH plus ProAgio. One-way ANOVA was used to determine *p*-values. Error bars indicate SD. ns, non-significant, $**p < 0.01$, $***p < 0.001$, and $****p < 0.0001$.

Supplementary figure 2: (A). IHC images showed increased CD68 **(A)** and COL1A1 **(B)** expression. IHC quantifications for CD68 **(C)** and COL1A1 **(D)**. One-way ANOVA was used to determine *p*-values. Error bars indicate SD. ns, non-significant, $*p < 0.05$, and $***p < 0.001$.

Supplementary Figure 3: Gating strategy used for the multiparameter flow cytometry.
